# Solution-Processed Organic Photovoltaics Based on Indoline Dye Molecules Developed in Dye-Sensitized Solar Cells

**DOI:** 10.3390/molecules18033107

**Published:** 2013-03-07

**Authors:** Zhaoyang Liu, Haruhiko Ojima, Ziruo Hong, Junji Kido, Wenjing Tian, Xiao-Feng Wang

**Affiliations:** 1 Department of Organic Device Engineering, Research Center for Organic Electronics, Yamagata University, Yonezawa, Yamagata 992-8510, Japan; 2 State Key Laboratory of Supramolecular Structure and Materials, Jilin University, Changchun 130012, China

**Keywords:** solution processed, indoline dye, organic solar cells, dye-sensitized solar cells

## Abstract

A donor-acceptor (D-A) type indoline dye, D149, was used as an electron donor in solution-processed organic solar cells (OSCs). For bulk-heterojunction (BHJ) type OSCs with PC_70_BM as electron acceptor, the power conversion efficiency (PCE) is sensitive to the amount of D149 in the D149/PC_70_BM blend film. When the concentration of D149 in the blend film was as low as 5%, the highest PCE of up to 1.29%, together with a short-circuit current density (*Jsc*) of 4.58 mA·cm^−2^, an open-circuit voltage (*Voc*) of 0.90 V and a fill factor (FF) of 0.31, was achieved. In order to improve the PCE of D149-based OSCs, a bilayer-heterojunction configuration with C_70_ as electron acceptor has been employed. By optimizing the thickness of the D149 layer and varying the electron- and hole-transport layers, a highest PCE of up to 2.28% with a *Jsc* of 4.38 mA·cm^−2^, a *Voc* of 0.77 V, and an FF of 0.62 was achieved under AM 1.5G solar illumination (100 mW·cm^−2^).

## 1. Introduction

Organic solar cells (OSCs) have been regarded as one of the major alternatives to the traditional silicon-based photovoltaics, owning to their potential low-cost and short energy pay-back time [1,2,3,4]. In OSCs, the photon-induced charge carriers are always generated at the interface between the p-type electron donor and n-type electron acceptor, and thus is followed by complicated charge transfer processes. Not only the photocurrent, but also the photovoltage of OSCs are mainly determined by the type of materials used in the p-n heterojunction. Therefore, development of effective n-type and p-type materials is the key to improve the photovoltaic performance of OSCs. Unlike most n-type materials that focus on fullerene and their derivatives, p-type materials offer more variability, including polymers and small molecules, and the highest power conversion efficiencies (PCE) for OSCs based on polymers and small molecules have been continuously improved [5,6,7,8,9,10].

Compared to polymers, organic small molecule dyes offer potential advantages such as ease of synthesis and purification, and a great variety of tailored structures to satisfy the energy-level alignments or optical properties within the device. Moreover, the D-A type of molecular architecture is very effective at producing low band-gap materials where the HOMO and LUMO levels can be controlled and this has become a common approach for the design of materials with broad absorption for use in organic solar cells. It is also a very effective way to tailor molecular donors as evidenced by the numerous reports on this type of material published in recent years [11,12].

In view of this, the indoline dye D149 (5-[[4-[4-(2,2-diphenylethenyl)phenyl]-1,2,3,3a,4,8b-hexahydrocyclopent[b]indol-7-yl]methylene]-2-(3-ethyl-4-oxo-2-thioxo-5-thiazolidinylidene)-4-oxo-3-thiazolidineacetic acid) is a very promising p-type donor candidate in OSCs. It has been proved that this dye has a significantly high molar extinction coefficient and the capability to efficiently transfer electrons to TiO_2_ during the charge-separation step; dye-sensitized solar cells (DSCs) based on such indoline sensitizer dyes can give PCEs of more than 9% [13,14,15,16,17,18]. However, some drawbacks, which prevent commercial applications of D149 in DSCs, such as electrolyte leakage and lack of flexibility, still need to be overcome. D149-based DSCs with solid-state hole-transporters instead of electrolytes could solve the problems of electrolyte leakage and electrode corrosion, but other problems such as high fabrication costs and poor reproducibility remain challenging [19,20,21].

In this study, we report the D149 dye as an electron donor for solution-processed OSCs. To the best of our knowledge, this is the first time that a highly efficient DSC dye sensitizer has been directly employed as the electron donor for both bulk- and bilayer-heterojunction OSCs.

## 2. Results and Discussion

### 2.1. Molecular Structure

**Figure 1:**
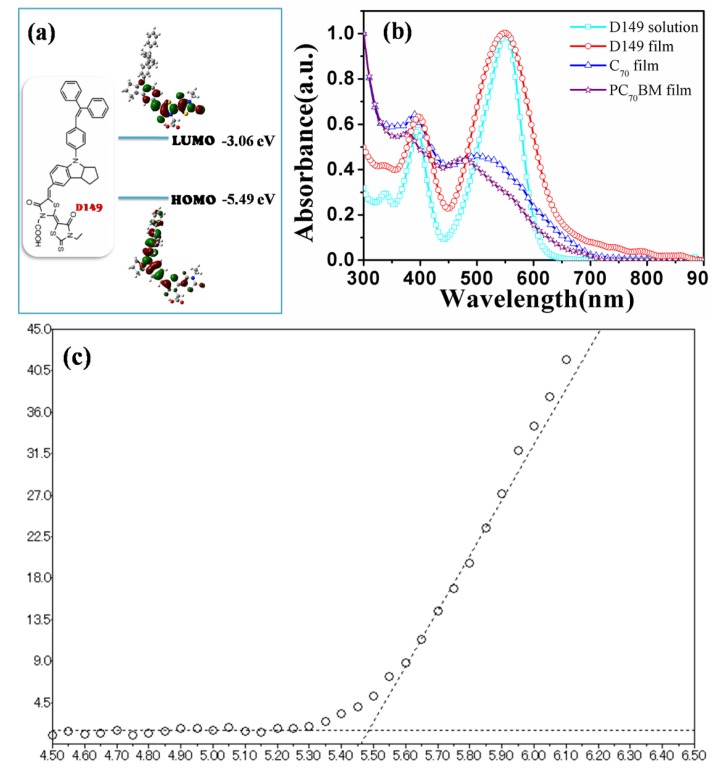
(**a**) Molecular structure and molecular orbitals of D149; (**b**) Normalized Absorption spectra of D149 in solid film and solution, and C_70_ and PC_70_BM in solid films; (**c**) The HOMO level of D149 measured by AC-3 was −5.49 eV.

The molecular structure of D149 is shown in Figure 1a. D149 consists of a D-A structure, in which the indoline unit acts as an electron-donating moiety and the carboxyl-functionalized double rhodanine rings act as electron-withdrawing moieties. Figure 1a also shows the calculated HOMO and LUMO of D149 by density functional theory (DFT), together with the energy levels obtained from UV photoelectron spectroscopy. The Photoelectron Yield Spectrometer AC-3 setup was utilized to measure the HOMO level of D149. The HOMO energy level of D149 measured in thin solid film by AC-3 is −5.49 eV (Figure 1c). The LUMO energy level of D149 was calculated to be −3.06 eV from the measured HOMO level and the optical band-gap (2.43 eV) of D149 from its UV-Vis absorption spectrum. As a result of the D-A architecture of D149, intramolecular charge-transfer (ICT) could take place [22]. The electrons move from the indoline moiety in the highest occupied molecular orbital (HOMO) to the rhodanine moiety in the lowest unoccupied molecular orbital (LUMO). The HOMO level of D149 is deep enough for a build-in potential with a fullerene-type electron acceptor, while the LUMO level is high enough for efficient interfacial charge transfer to PC_70_BM.

Figure 1b shows the normalized ultraviolet-visible (UV-Vis) absorption spectra of D149 dye in CHCl_3_ solution and spin-cast as a thin solid film, and compared to the absorption spectrum of C_70_ and PC_70_BM films. The broader absorption peaks of D149 in the thin solid film than in solution could be attributed to aggregate formation as a result of fast solvent evaporation, although this is not as serious as in DSCs. It has been found that D149 is more soluble in CHCl_3_ than in other typical OSC solvents. All subsequent characterizations and solar cell device fabrications were therefore carried out using CHCl_3_ solutions of D149. The absorption spectra for the donor D149 and the acceptor C_70_ and PC_70_BM are quite similar in the absorption region, but with differences in the density of each absorption peak.

### 2.2. BHJ Solar Cells Based on a D149/PC_70_BM

**Figure 2:**
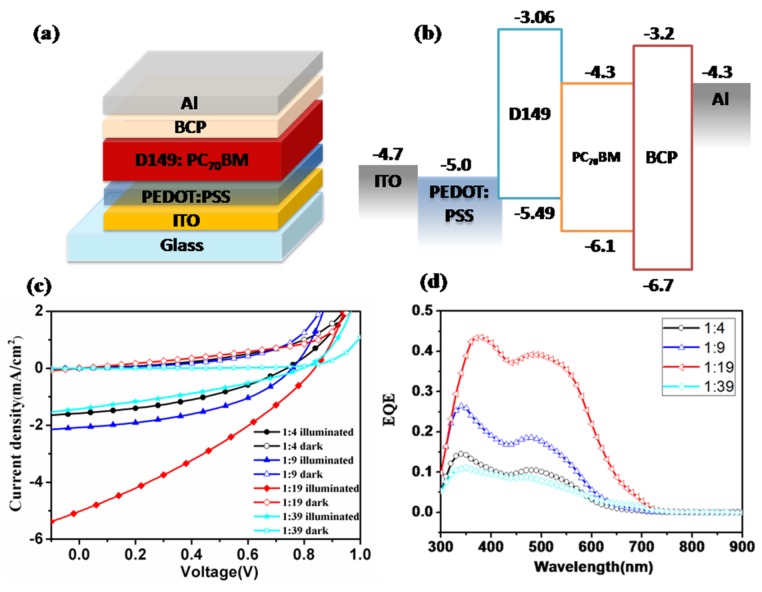
(**a**) Device architecture of BHJ solar cell, ITO/PEDOT: PSS/D149: PC_70_BM/BCP/Al; (**b**) Energy level alignment within the BHJ device; (**c**) *J-V* curves of the BHJ solar cell with different blending ratios (w/w) of D149: PC_70_BM; (**d**) EQE spectra of the corresponding BHJ devices.

In order to produce a large interface between the donor and acceptor molecules, solution-processed bulk-heterojunction (BHJ) devices based on a D149/PC_70_BM active layer were fabricated first. Figure 2a shows the device structure, *i.e.*, ITO/PEDOT: PSS (40 nm)/D149:PC_70_BM/BCP (10 nm)/Al (100 nm). PEDOT: PSS acts as a hole-transporting layer and BCP acts as an exciton-blocking layer. The active layer was prepared by spin-coating CHCl_3_ solutions containing 1 mg·mL^−1^ of D149 with different amounts of PC_70_BM, to give a final D149 concentration of 2.5–20 wt% in the blend film. Figure 2b shows the energy level alignment of different materials in the BHJ solar cells. Both the HOMO (−5.49 eV) and LUMO level (−3.06 eV) of D149 are above that of PC_70_BM, indicating that hot-electron transport from D149 to PC_70_BM is favorable.

Figure 2c,d shows the current density-voltage (*J-V*) curves and external quantum efficiencies (EQE) spectra of the BHJ cells based on different D149/PC_70_BM blending ratios. Table 1 lists the relevant parameters extracted from the *J-V* curves. Interestingly, the best PCE obtained for such a BHJ device was 1.29%,with a *Voc* of 0.90 V, a *Jsc* of 4.58 mA·cm^−2^, and a FF of 0.31, for D149/PC_70_BM with a blending ratio as low as 1:19 (5% weight content of D149). This suggests that a low concentration of donor molecules in the blend film may play a different role in such a cell from that in other BHJ OPVs. Similar phenomena have already been observed for thermally evaporated BHJ devices, in which concentrations of donor molecules as low as 5% gave the highest PV performance. The mechanism has been partially attributed to Schottky barrier contact between the donor and MoOx layers [23], however, this is obviously not the case in our system, since replacing PEDOT:PSS with MoO_3_ gave a slightly lower PCE of 1.02%, with a *Voc* of 0.90 V, a *Jsc* of 3.72 mA·cm^−2^, and an FF of 0.31. The difference in PCE originated mainly from the different *Jsc* values of the two devices. The HOMO level of D149 is higher than the work function of MoO_3_ (−5.50 eV), and lower than the work function of PEDOT: PSS (−5.00 eV). Hole injection from D149 to PEDOT: PSS will therefore be more effective than that to MoO_3_, which would result in a higher *Jsc* of the device. On the other hand, the relatively low FF of both devices might be attributed to poor charge-transport within the active layers. The high polarity of the D149 dye not only causes the low solubility of the dye in organic solvents, but also reduces the charge mobility of the molecule and the resultant carrier-transport in blend films. In order to achieve better PV performances, it is important to overcome the issue of the low carrier-mobility of the D149 donor molecule in OPV devices. Fabrication of a bilayer-heterojunction device with a relatively thin layer of D149 is therefore preferable, since charge transport in such a device might be less affected by the charge-carrier mobility.

**Table 1:** Performance details of the photovoltaic devices sharing a structure of ITO/PEDOT: PSS/D149:PC_70_BM/BCP/Al, varying the blending ratios (w/w) of D149: PC_70_BM from 1:4 to 1:39, under AM 1.5G 100 mW·cm^−2^ illumination.

**Blending ratio** (w/w) of D149:PC_70_BM	***Jsc* (mA/cm^2^)**	***Voc* (V)**	**FF**	**PCE (%)**
**1:4**	1.58	0.74	0.39	0.45
**1:9**	2.08	0.76	0.44	0.69
**1:19**	4.58	0.90	0.31	1.29
**1:39**	1.42	0.80	0.32	0.36

### 2.3. Bilayer-Heterojunction Solar Cells based on a D149/C_70_

**Figure 3:**
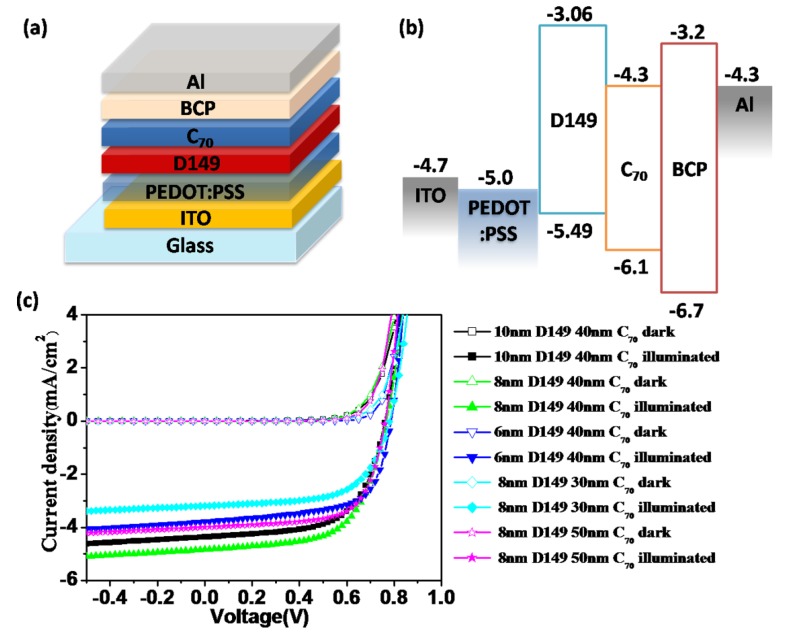
(**a**) Device architecture of bilayer devices; (**b**) Energy level alignment within the devices; (**c**) *J-V* curves of the bilayer devices with different thickness of D149 and C_70_.

Figure 3a shows a bilayer-heterojunction PV device with the structure ITO/PEDOT:PSS (30 nm)/D149/C_70_ (40 nm)/BCP (10 nm)/Al (100 nm), and Figure 3b shows the energy alignment of each layer. D149 layers of 6–10 nm thickness were obtained by spin-coating a 1 mg·mL^−1^ D149 CHCl_3_ solution on top of the PEDOT: PSS layer. The best PV performance for this bilayer structure was 2.28% for a PCE calculated from a *Voc* of 0.77 V, a *Jsc* of 4.83 mA·cm^−2^, and an FF of 0.62, using an 8 nm-thick D149 layer. The PV performance decreases for both thinner and thicker D149 layers, as shown in Table 2. More specifically, the PCE of the reference device with a 10 nm-thick D149 layer was 2.06%, with a *Voc* of 0.76 V, a *Jsc* of 4.36 mA·cm^−2^, and an FF of 0.62, whereas a PCE of 1.93%, with a *Voc* of 0.78 V, a *Jsc* of 3.83 mA·cm^−2^, and an FF of 0.65 was obtained for a device based on a 6 nm-thick D149 layer. In the best BHJ device described above, the enhanced values of *Jsc* and FF for the bilayer structure device could both be attributed to facilitated hole-transport in such a thin film of D149, since the thickness of the D149 layer is within the resonance energy transfer distance. Obviously, the reduced PV performance with increased film thickness from 8 nm to 10 nm also supports the above theory, especially for donor molecules with relatively low carrier-mobility. On the other hand, the decreased PV performance with decreased film thickness from 8 nm to 6 nm was mainly the result of reduced photocurrent generation, originating from the low light-harvesting capabilities of such OPV devices. The better photovoltaic performance of D149 in bilayer-heterojunction devices than in BHJ devices was due to its very high light absorption coefficients so that 8–10 nm thick dye layer can capture most of the visible light, maintaining the effective hole transportation within such a thin layer at the same time.

**Table 2:** Performance details of the photovoltaic devices sharing a structure of ITO/PEDOT: PSS/D149/C_70_/BCP/Al, varying the thickness of D149 from 6 nm to 10 nm and the thickness of C_70_ from 30 nm to 50 nm, under AM 1.5G 100 mW·cm^−2^ illumination.

Layer thickness	*Jsc* (mA/cm^2^)	*Voc* (V)	FF	PCE (%)
**D149 6 nm C_70_ 40 nm**	3.83	0.78	0.65	1.93
**D149 8 nm C_70_ 40 nm**	4.83	0.77	0.62	2.28
**D149 10 nm C_70_ 40 nm**	4.36	0.76	0.62	2.06
**D149 8 nm C_70_ 30 nm**	3.19	0.78	0.62	1.55
**D149 8 nm C_70_ 50 nm**	4.00	0.76	0.67	2.04

Similarly, the thickness of the C_70_ layer has also been optimized for OPV devices with an 8 nm-thick D149 layer. The device with a 30 nm-thick C_70_ layer gave a PCE of 1.55%, with a *Voc* of 0.78 V, a *Jsc* of 3.19 mA·cm^−2^, and an FF of 0.62. The device with a 50 nm-thick C_70_ layer had a PCE of 2.04%, with a *Voc* of 0.76 V, a *Jsc* of 4.00 mA·cm^−2^, and an FF of 0.67. The changes in PV performance for different C_70_ thicknesses are caused mainly by changes in the photocurrent. The C_70_ thickness was changed in steps of 10 nm, and this could cause large differences in the optical effects within the device and in photon capture within the active layer.

**Figure 4:**
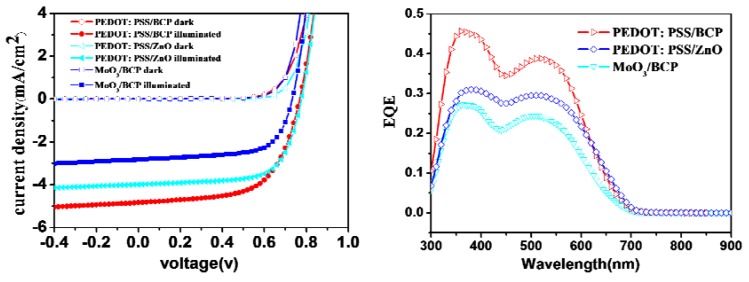
*J-V* curves and EQE profiles of devices based on a structure of ITO/Hole transport layer/D149 (8 nm)/C_70_ (40 or 50 nm)/Electron transport layer/Al (100 nm) with different electron- and hole-transport layers under AM 1.5G illumination or dark conditions.

The electron- and hole-transport layers on both sides of the bilayer device are another important issue in determining the PV performances of such OPVs. We chose ZnO and MoO_3_ as alternatives to BCP and PEDOT: PSS for the electron- or hole-transport layers. Figure 4 shows the *J-V* curves and external quantum efficiency (EQE) spectra of OPV devices based on different electron- and hole-transport layers. Table 3 lists detailed parameters extracted from the *J-V* curves. Unlike the BCP layer, which was evaporated onto the C_70_ layer, the ZnO layer was spin-coated from a ZnO nanoparticle suspension. A 40 nm-thick C_70_ layer in the best OPV device was found to be insufficient to protect the D149 under-layer from a ZnO/butanol solution. A 50 nm-thick C_70_ layer was therefore used instead. Actually, there has been none of example of using ZnO as the electron transport layer for the bilayer-heterojunction OSC devices, since transferring samples out of vacuum chamber before depositing metal electrode usually causes very bad PV performance. Therefore, the success of using solution processed ZnO electron transport layer in the present study newly provided a useful tool for improving the PV performance of thermal evaporated OSC devices. The OSC device based on a solar cell configuration of ITO/PEDOT:PSS (40 nm)/D149/C_70_ (50 nm)/ZnO (10 nm)/Al (100 nm) gave a PCE of 2.10%, with a *Voc* of 0.78 V, a *Jsc* of 3.99 mA·cm^−2^, and an FF of 0.68; this was slightly worse than the PCE of a PEDOT:PSS/BCP-based OPV device with a 40-nm-thick C_70_ layer, but better than that with a 50-nm-thick C_70_ layer. The high FF in the ZnO-based device could be attributed to the excellent electron-mobility of ZnO, which reduces the device resistance. In contrast, the photocurrent and solar cell performance were substantially lowered by replacing PEDOT: PSS with MoO_3_. This might be caused by a poorer interface between the MoO_3_ and D149 layers. Moreover, the fact that the *Jsc* values in the *J-V* curves are in good agreement with the integrated area under the external quantum efficiency profiles strongly supports our statement above.

**Table 3:** Photovoltaic performances of D149 based bilayer OPV devices based on a structure of ITO/Hole transport layer/D149 (8 nm)/C_70_/Electron transport layer/Al using different electron- and hole-transport layers.

Hole/Electron transport layer	*Jsc* (mA/cm^2^)	*Voc* (V)	FF	PCE (%)
**PEDOT: PSS/BCP**	4.83	0.77	0.62	2.28
**PEDOT: PSS/ZnO**	3.99	0.78	0.68	2.10
**MoO_3_/BCP**	2.83	0.73	0.65	1.36

**Figure 5:**
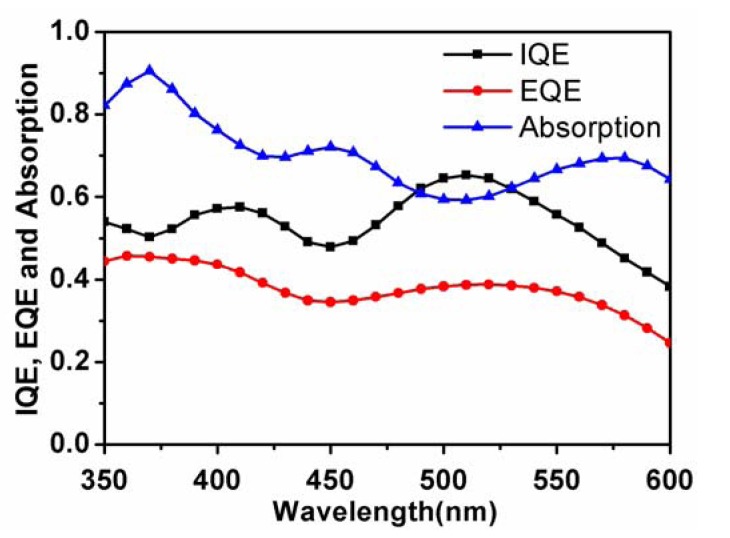
IQE, EQE and normalized absorption spectra for the bilayer device with the structure ITO/PEDOT: PSS (30 nm)/D149 (8 nm)/C_70_ (40 nm)/BCP (10 nm)/Al (100 nm).

Finally, it is a question that whether the low photocurrent of D149 based solar cells comes from the limitation of charge separation and collection processes. In Figure 5, we measured the absorption of the D149 based bilayer device, and calculated the internal quantum efficiency (IQE) by the use of the observed EQE spectrum. In a broad region from 350–600 nm, the IQE values of the solar cell were more than 40%. The light-harvesting capability of the solar cell contributes ~20% of the energy loss, while the other 30%–40% comes from the charge separation and charge transport within the solar cell device. Thus, further improvement of the carrier mobility of the indoline dye should be the key to improve the OPV performance. Moreover, about the charge separation mechanism in the present system, the clear EQE response from the absorption region of the D149 absorption peaks strongly suggests that the hot carriers was transferred to the acceptor molecules from the optically-transition-allowed electronic state of D149, as suggested in the investigation on carotenoid-based OSCs [24].

## 3. Experimental

### 3.1. Materials

D149 was purchased from Mitsubishi Paper Mills Ltd. (Tokyo, Japan) and used without any treatment. PC_70_BM and C_70_ were purchased from Luminescence Technology Corp. (Taipei, Taiwan) and C_70_ was used after being sublimed four times. Sublimated 2, 9-dimethyl-4, 7-diphenyl-1, 10-phenanthroline (BCP) was purchased from Wako Chemicals (Wako, Japan). ZnO-350 nanoparticles received from Sumitomo Osaka Cement Co.Ltd, (Osaka, Japan) (cast from butanol solution, 10 mg·mL^−1^). Poly (3, 4-ethylenedioxythiophene): poly (styrenesulfonate) (PEDOT: PSS) aqueous solution was purchased from H. C. Starck (Leverkusen, Germany, Clevios P VP.AI 4083).

### 3.2. Device Fabrication and Characterization

For device fabrication, a patterned ITO coated glass substrate was cleaned by sequential ultrasonic treatment in detergent deionized water, acetone, and isopropyl alcohol for 30 min, respectively, then treated by UV-ozone for 30 min, followed by spin-coating a thin layer of PEDOT:PSS from aqueous solution. Then the PEDOT: PSS coated substrate was annealed for 30 min at 140 °C in air. Solution processed BHJ devices with ITO/MoO_3_ (5 nm) or PEDOT: PSS (40 nm)/D149:PC_70_BM/BCP (10 nm)/Al (100 nm) structures were fabricated. The solutions were made by adding 4, 9, 19, and 39 mg PC_70_BM to D149 solutions (1 mL, 1 mg·mL^−1^) respectively, and stirring overnight. The active layer was spin-coated on top of the PEDOT: PSS or MoO_3_ layer at a speed of 5,000 rpm, followed by vacuum deposition of BCP and Al, respectively. Bilayer-heterojunction solar cells with a structure of ITO/PEDOT: PSS (30 nm)/D149/C_70_/BCP (10 nm)/Al (100 nm) were fabricated. A thin layer of D149 (6, 8 and 10 nm, respectively) was spin coated on top of the PEDOT: PSS layer in a nitrogen glove box, followed by vacuum deposition of C_70_ (30, 40 and 50 nm, respectively), BCP, and Al. Both PCE and EQE characterizations of photovoltaic cells were carried out on a CEP-2000 integrated system by Bunkoukeiki Co. (Tokyo, Japan) under standard measurement conditions [25].

## 4. Conclusions

In conclusion, we have demonstrated a strategy for using a typical DSC dye, namely D149, to fabricate solution-processed bulk- and bilayer-heterojunction OSCs. The D149 bilayer devices give higher PV performances than BHJ devices. The thicknesses of the donor and acceptor layers in the bilayer device can substantially affect the OPV performance. Optimization of the electrode-modification materials achieves a highest PCE of 2.28%. The present study provides some scope for alternative uses of the dye sensitizers discovered in current DSC studies.
